# A Real-Time Neurophysiologic Stress Test for the Aging Brain: Novel Perioperative and ICU Applications of EEG in Older Surgical Patients

**DOI:** 10.1007/s13311-023-01401-4

**Published:** 2023-07-12

**Authors:** Miles Berger, David Ryu, Melody Reese, Steven McGuigan, Lisbeth A. Evered, Catherine C. Price, David A. Scott, M. Brandon Westover, Roderic Eckenhoff, Laura Bonanni, Aoife Sweeney, Claudio Babiloni

**Affiliations:** 1 Department of Anesthesiology, Duke University Medical Center, Duke South Orange Zone Room 4315B, Box 3094, Durham, NC 27710 USA; 2 Duke Aging Center, Duke University Medical Center, Durham, NC USA; 3 Duke/UNC Alzheimer’s Disease Research Center, Duke University Medical Center, Durham, NC USA; 4 School of Medicine, Duke University, Durham, NC USA; 5 Department of Anaesthesia and Acute Pain Medicine, St Vincent’s Hospital, Melbourne, VIC Australia; 6 Department of Critical Care, School of Medicine, University of Melbourne, Melbourne, Australia; 7 Weill Cornell Medicine, New York, NY USA; 8 Clinical and Health Psychology, University of Florida, Gainesville, FL USA; 9 Norman Fixel Institute for Neurological Diseases, University of Florida, Gainesville, FL USA; 10 Department of Neurology, Beth Israel Deaconess Hospital, Boston, MA USA; 11 Department of Anesthesiology and Critical Care, Perelman School of Medicine, University of Pennsylvania, Philadelphia, PA USA; 12 Department of Medicine and Aging Sciences, University G d’Annunzio of Chieti-Pescara, Chieti, Italy; 13 School of Medicine, Dentistry and Biomedical Sciences, Queen’s University Belfast, Belfast, UK; 14 Department of Physiology and Pharmacology “Vittorio Erspamer”, Sapienza University of Rome, Rome, Italy; 15 San Raffaele of Cassino, Cassino, FR Italy

**Keywords:** Delirium, Anesthesia, Dementia, Alzheimer’s disease, Neurophysiology, Cognitive impairment

## Abstract

As of 2022, individuals age 65 and older represent approximately 10% of the global population [1], and older adults make up more than one third of anesthesia and surgical cases in developed countries [2, 3]. With approximately > 234 million major surgical procedures performed annually worldwide [4], this suggests that > 70 million surgeries are performed on older adults across the globe each year. The most common postoperative complications seen in these older surgical patients are perioperative neurocognitive disorders including postoperative delirium, which are associated with an increased risk for mortality [5], greater economic burden [6, 7], and greater risk for developing long-term cognitive decline [8] such as Alzheimer’s disease and/or related dementias (ADRD). Thus, anesthesia, surgery, and postoperative hospitalization have been viewed as a biological “stress test” for the aging brain, in which postoperative delirium indicates a failed stress test and consequent risk for later cognitive decline (see Fig. 3). Further, it has been hypothesized that interventions that prevent postoperative delirium might reduce the risk of long-term cognitive decline. Recent advances suggest that rather than waiting for the development of postoperative delirium to indicate whether a patient “passed” or “failed” this stress test, the status of the brain can be monitored in real-time via electroencephalography (EEG) in the perioperative period. Beyond the traditional intraoperative use of EEG monitoring for anesthetic titration, perioperative EEG may be a viable tool for identifying waveforms indicative of reduced brain integrity and potential risk for postoperative delirium and long-term cognitive decline. In principle, research incorporating routine perioperative EEG monitoring may provide insight into neuronal patterns of dysfunction associated with risk of postoperative delirium, long-term cognitive decline, or even specific types of aging-related neurodegenerative disease pathology. This research would accelerate our understanding of which waveforms or neuronal patterns necessitate diagnostic workup and intervention in the perioperative period, which could potentially reduce postoperative delirium and/or dementia risk. Thus, here we present recommendations for the use of perioperative EEG as a “predictor” of delirium and perioperative cognitive decline in older surgical patients.

An irony of perioperative medicine is that although the central nervous system (CNS) is the target of most if not all anesthetic/analgesic drugs, the CNS is the only major organ system that is not typically evaluated prior to surgery, nor routinely monitored during anesthetic care in the USA. The American Society of Anesthesiology (ASA) calls for preoperative assessment of cardiac and pulmonary function [9], and intraoperative evaluation of blood pressure, heart rate and pulse oximetry [10], yet these guidelines make no mention of evaluating the brain (or the CNS more broadly) before surgery nor of monitoring it during surgery. While unfortunate, these omissions are logical from the perspective of intraoperative care. Unlike cardiac findings such as critical aortic stenosis that would lead to specific changes in intra- and peri-operative anesthetic management, there is currently no evidence-based anesthetic management approach to improve outcomes for individuals with preoperative cognitive impairment.

Although not required by ASA guidelines, intraoperative electroencephalography (EEG) is used by many anesthesiologists to titrate anesthetic drug administration. Several frontal EEG electrode systems are commercially available and used widely in the USA, Europe, Asia, and Australia. These frontal electrode arrays have predominantly been marketed for use in anesthetic titration to ensure avoidance of intraoperative recall (i.e., to facilitate intraoperative amnesia) while facilitating rapid emergence from the anesthetized state. However, their ability to achieve the former has been questioned [11]. Yet, an emerging body of evidence in both human neuroscience and perioperative medicine suggests that these monitors may be useful for a different purpose: identifying older patients with atypical waveforms indicative of underlying risk for neuropathologies such as postoperative delirium and/or long-term cognitive decline and dementia [12]. In this article, we review both traditional applications and these novel potential uses of perioperative EEG monitoring, and we discuss how they might be used in the future.

## Awake/Resting-State EEG Assessment of Neurocognitive Vulnerability to ADRD

Aside from using EEG for intraoperative anesthetic titration (and/or for attempting to prevent intraoperative awareness with explicit recall), frontal EEG monitors can be placed prior to anesthetic induction, either in a preoperative holding area or in the operating room. Although most anesthesiologists do not formally record baseline drug-free raw EEG signals or processed EEG values prior to anesthetic induction, placement of EEG monitors before induction provides this opportunity. Further, the fact that patients with dementia have altered preoperative processed EEG values [13] demonstrates that preoperative EEG contains information about the baseline status of brain health in our patients.

Indeed, the idea that resting-state EEG contains information about brain structure and function has been heavily explored in the neurology and neurodegenerative disease fields, partly due to the unmet need for non-invasive, low-cost biomarkers sensitive to preclinical stages (including structural markers) of aging-related neurodegenerative disorders such as Alzheimer’s (AD), Parkinson’s (PD), Lewy body dementia (LBD), and other types of dementia [14–16]. Promising candidate electrophysiologic biomarkers have been derived from eyes-closed resting-state EEG rhythms, which may reflect the neurophysiological dysregulation of quiet vigilance (i.e., level of consciousness) in people with AD [17–19]. Indeed, most people with AD struggle to maintain adequate vigilance while transitioning to drowsiness when watching a TV program or during a quiet social conversation [20].

**Fig. 1:**
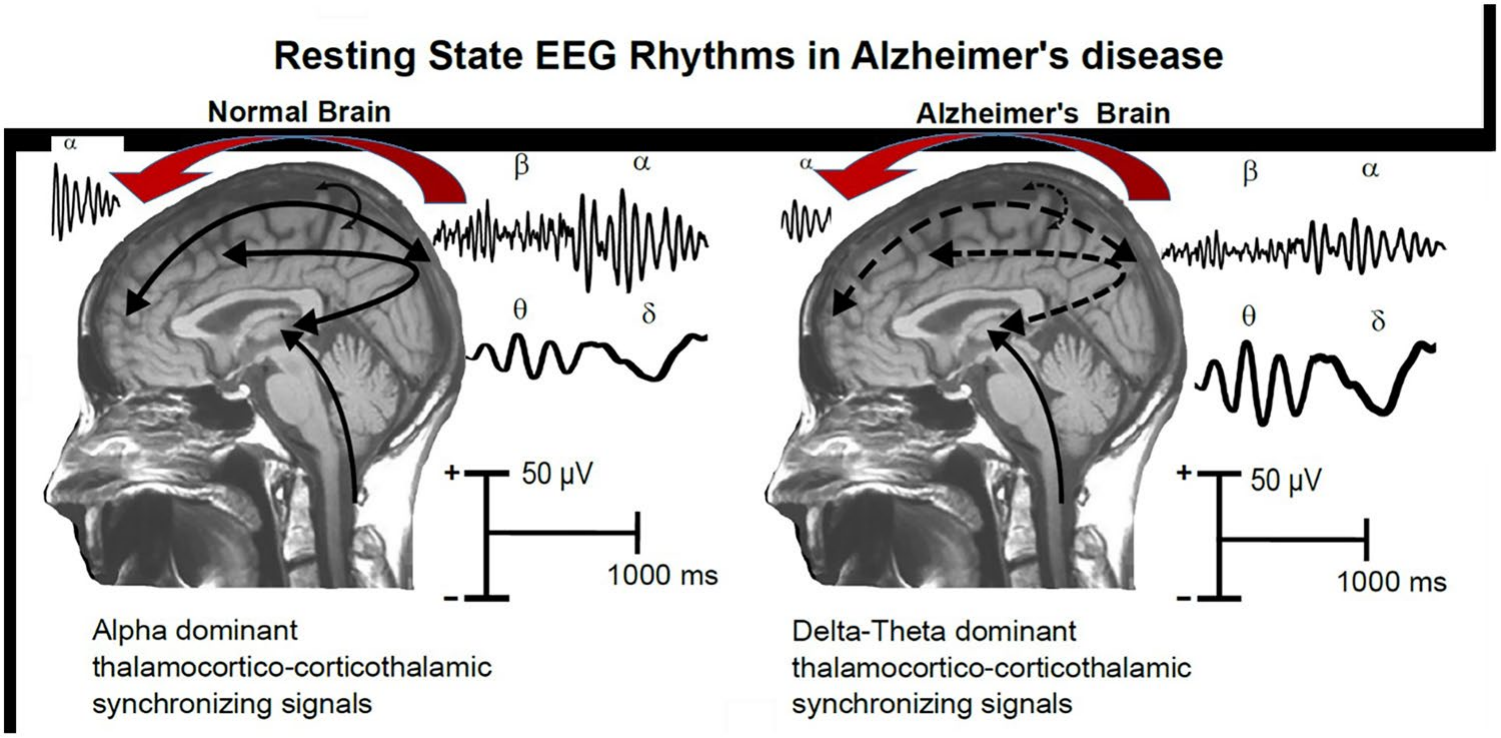
A tentative physiological model of resting state EEG oscillations in the normal and Alzheimer’s disease brain, in the awake state and in the anesthetized state. The red arrows indicate anteriorization of the EEG rhythms due to GABAergic anesthetic agents such as propofol or volatile anesthetics. In the normal brain, dominant EEG rhythms are observed at alpha frequencies (8–12 Hz), denoting the background, spontaneous synchronization ~ 10 Hz of neural networks that regulate global arousal and consciousness states. These networks span neural populations of the cerebral cortex, thalamus, basal forebrain, and brainstem, including glutamatergic, cholinergic, dopaminergic, and serotoninergic parts of the reticular ascending systems. The “slowing” of rsEEG rhythms depicted in the Alzheimer’s brain would mainly reflect a thalamocortical “disconnection.” Adapted from Babiloni et al., 2021 (courtesy of the Publisher) [19]

In these resting-state EEG studies, participants typically sit in a silent, dimly lit room. Participants are asked to remain awake, to allow their minds to wander freely, and to abstain from any specific cognitive tasks. The resting-state EEG recording is typically performed for 3–5 min with eyes closed and 3–5 min with eyes open to manipulate quiet vigilance with differing levels of visual input. Overall, resting-state EEG recordings are non-invasive, affordable and available worldwide [21]. After the resting-state EEG recording, power (amplitude^2^) can be calculated from artifact-free ongoing EEG activity within specific frequency bands, such as delta (< 4 Hz), theta (4–7 Hz), alpha (8–12 Hz), beta (13–35 Hz), and gamma (35–45 Hz). The topographical (or spatial) maps of the power of resting-state EEG rhythms are thought to reflect the cerebral neurophysiological oscillatory mechanisms underpinning cortical arousal as a balance of neural excitation/inhibition in (1) neuromodulatory subcortical ascending activating systems, (2) thalamocortical-corticothalamic loops including thalamocortical relay-mode and reticular thalamic neuronal populations, and (3) cortico-cortical circuits [17, 22, 23] (see Fig. 1).

**Fig. 2:**
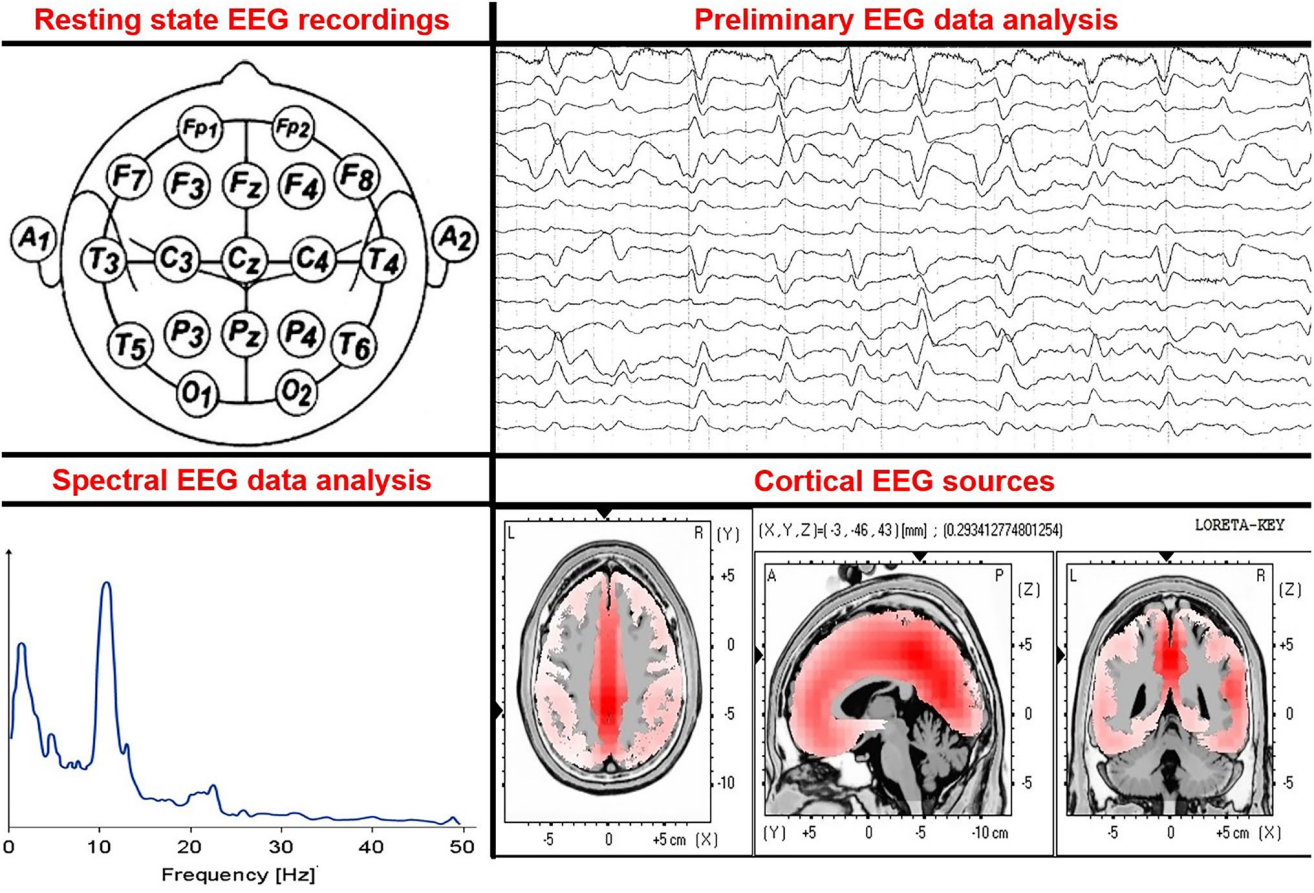
An overview of electroencephalographic (EEG) methodology to investigate cortical rhythm changes related to altered vigilance in older adults at risk of postoperative delirium and/or mild cognitive impairment due to age-related neurodegenerative diseases. Top left: top down view of 19 scalp electrodes from the International 10–20 System (Fp1, Fp2, F7, F3, Fz, F4, F8, T3, C3, Cz, C4, T4, T5, P3, Pz, P4, T6, O1, and O2). This setup has been used for many rsEEG recordings. In this figure, A1 and A2 indicate the position of linked earlobe reference electrodes. Top right: example of resting-state electroencephalographic (rsEEG) activity with artifacts. Bottom left: an example of an EEG power density spectrum computed at an occipital electrode (i.e., O1) in a healthy control person. Note the EEG power density peak at 10 Hz is typically called “individual alpha frequency peak (IAFp),” which can be used to distinguish low- and high-frequency alpha sub-bands within individuals. Bottom right: an example of cortical source solutions from rsEEG alpha rhythms computed by the exact low-resolution brain electromagnetic tomography (eLORETA) freeware. The eLORETA source solutions can be averaged within cortical lobes of interest and compared among older adults with intact cognitive status (including those with preclinical Alzheimer’s disease (AD) pathology) and individuals with AD and related dementias

In cognitively normal healthy adults with their eyes closed, the greatest resting-state EEG signal power occurs within 8–12 Hz (i.e., the alpha rhythm) over parietal and occipital scalp regions, which is thought to reflect cortical inhibition in the posterior visual-spatial cortical areas during relaxation and quiet vigilance (see Fig. 2, bottom left panel). Aside from these alpha rhythms, there is little power in other resting-state EEG frequencies in cognitively normal individuals with their eyes closed [17]. When individuals open their eyes, visual information processing in posterior visual areas is accompanied by increased cortical excitation in these areas with a mild increase in vigilance level, which is seen on resting-state EEG as a reduced amplitude of the posterior EEG alpha rhythms [24]. This phenomenon is referred to as a desynchronization or block of the posterior alpha rhythms, and these sensory stimuli induce transient increases in EEG power in delta, theta, beta, and gamma frequency bands [17–19, 22].

Previous studies have also investigated early abnormalities in resting-state EEG rhythms in patients with MCI, AD, and subjective memory/cognitive complaints (SMC). Compared with cognitively normal individuals, patients with MCI and/or AD showed (1) greater power or source activation in spatially widespread resting-state EEG delta and theta rhythms, (2) lower power or source activation in posterior resting-state EEG alpha and beta rhythms, and (3) lower resting-state EEG alpha peak frequency [25–28] (see Fig. 1). Those abnormalities were related to structural magnetic resonance imaging (MRI) markers of cortical neurodegeneration [29]. Furthermore, those EEG changes were more prominent in carriers of independent AD risk factors such as the cystatin C [30] and ApoE ε4 alleles [31].

Other studies have investigated the “interrelatedness” of resting-state EEG rhythms between electrode or source pairs, via measures such as coherence (the temporal synchronicity of two brain regions), synchronization likelihood (a measure of both linear and non-linear coupling), and/or lagged linear connectivity (a computed estimate of functional cortical connectivity), as models of dysfunctional cortical connectivity in AD patients [32]. For instance, both MCI and AD patients showed abnormally increased delta and reduced resting-state EEG alpha “interrelatedness,” both intra-hemispherically and inter-hemispherically [33, 34]. Similarly, these patients showed decreased parietal-to-frontal “interrelatedness” of resting-state EEG alpha rhythms in association with subcortical white-matter lesions [35], and decreased global “interrelatedness” of EEG alpha rhythms with impairment of ascending cholinergic tracts [36].

Furthermore, among individuals with MCI, abnormal cerebrospinal fluid (CSF) amyloid β42 levels have been associated with greater low-frequency (delta and theta) resting-state global field power (i.e., the standard deviation of EEG potentials across all electrodes), whereas abnormal phospho-tau and total tau levels have been associated with reduced mid-high frequency (alpha and beta) resting-state EEG global field power [37]. Similarly, when compared to cognitively normal older adults, people with MCI (and SMC) who were not tested for brain amyloid deposition still showed greater delta source activity and reduced posterior resting-state EEG alpha source activity [25]. For individuals with AD, abnormal p-tau and total tau values were associated with a reduction in low (delta) and high (beta) frequency global field power, which may suggest that alpha global field power is more specific to phospho-tau and total tau-related brain changes in MCI than in AD [37].

In the INSIGHT-preAD study, people with and without SMC underwent 18F-florbetapir positron emission tomography (PET) to detect brain amyloid deposition and also underwent resting-state EEG. There was only a very mild association between amyloid deposition and posterior resting-state EEG alpha power, while no effect was observed on the “interrelatedness” of resting-state EEG activity at electrode pairs [38]. High education level was associated with greater parietal, occipital and temporal resting-state EEG alpha power, and was associated with a “rescue” of the low posterior resting-state EEG alpha power in amyloid positive individuals with SMC [39].

Thus, numerous studies have shown significant changes in the topography and power of resting-state EEG rhythms in older patients with AD, PD, LBD, and other related dementias. A seminal review of resting-state EEG studies performed between 1980–2009 reported an average accuracy of 80% in differentiating patients with AD vs. cognitively normal individuals based on spectral resting-state EEG measures [40]. However, these authors raised the issue of a questionable diagnostic utility for routine clinical applications, as “classification accuracy” does not mean “diagnosis” [40]. For instance, in a systematic review of resting-state EEG studies in older patients with dementia, the Alzheimer’s Association ISTAART Electrophysiology Professional Interest Area (EPIA) did not recommend resting-state EEG biomarkers for AD diagnosis, since resting-state EEG biomarkers are not direct measures of brain amyloidosis or tauopathy. Rather, the ISTAART EPIA suggested using resting-state EEG for assessing abnormalities in the neurophysiological oscillatory systems regulating quiet vigilance in patients with MCI or AD, and in back-translational research in Alzheimer’s disease and related dementias (ADRD) mouse models [18, 19]. Similarly, the guidelines of the European Federation of the Neurological Societies (EFNS) state that resting-state EEG may provide supportive (but not diagnostic) information for assessing patients with ADRD [41–43]. In fact, most if not all clinical guidelines recommended the use of biomarkers directly reflecting neuropathology, neurodegeneration, and brain hypometabolism to diagnose patients with dementia; these guidelines either neither advocate for nor suggest against the use of EEG biomarkers for clinical diagnosis [44–48].

Thus, while several studies have successfully shown that spectral resting-state EEG measures computed at scalp electrodes or cortical sources can be used to predict cognitive decline over time in people with SMC, MCI, and ADRD (Table 1; Table 2 for data quality ratings), none of these resting-state EEG measures have sufficient sensitivity and specificity to be used for diagnosing ADRD. However, this literature demonstrates that there are resting-state EEG abnormalities across the spectrum of disease in ADRD that could be used as screening tests to identify at-risk patients. Further, since many of these EEG abnormalities can be detected in just minutes over frontal electrode sites [49–51] using relatively inexpensive equipment [21], anesthesiologists could use these frontal EEG patterns prior to surgery as screening tests to refer patients for further neuropsychologic/neurologic workup (and potential diagnosis) after they recover from surgery.

**Table 1:** Awake resting-state EEG findings across the ADRD spectrum

Study	Groups	EEG electrode type	Main Finding
Gouw et al., 2017 [52]	SCD (*n* = 63) vs MCI (*n* = 142)	21 electrodes 3 systems: Nihon Kohden digital EEG (EEG 2100; 2 versions of OSG digital equipment (BrainLab, BrainRT)	↑ Delta and theta power and ↓ alpha power and peak frequency associated with clinical progression from SCD -53% (*n* = 108) of subjects progressed from SCD to MCI/dementia or from MCI to dementia after 2.1 years
Prichep et al., 2006 [53]	Non-Decliners (*n* = 17), remained at SCD after ≥ 7 years vs Decliners (*n* = 27) who worsened to MCI or dementia	19 monopolar Ag/AgCl electrodes referenced to linked earlobes Brain State Analyzer (Cordis Corporation) or Spectrum 32 (Cadwell Laboratories)	Baseline ↑ theta power and slowing of mean frequency was seen in people with cognitive decline
Jelic et al., 2000 [54]	MCI progressed to clinically manifest AD (*n* = 14) MCI stable (*n* = 13) Healthy controls (*n* = 16) AD reference (*n* = 15)	20 electrodes, computer-based Bio-Logic Brain Atlas	-MCI subgroups and controls had no differences in baseline EEG -MCI subgroups had significantly↓baseline theta power compared to reference AD group -Posterior alpha and theta power combined with mean frequency predicted cognitive deterioration at 1 year follow up
Huang et al., 2000 [55]	Mild AD (*n* = 18) MCI (*n* = 31) Healthy controls (*n* = 24)	20 electrodes, computer-based Bio-Logic Brain Atlas	-Patients with AD had ↑ delta and theta GFP and ↓ alpha GFP compared to controls -Patients with AD had ↓ alpha and beta GFP compared to MCI subjects -Anteroposterior localization of alpha source activation was best predictor of future development of AD
Luckhaus et al., 2008 [56]	MCI (*n* = 88) Mild probable AD (*n* = 42)	32-Channel EEG	↓ Posterior alpha power was most highly predictive of MCI and AD, predicting cognitive decline by 1-year follow-up
Rossini et al., 2006 [57]	MCI (*n* = 69)	19 AgCl cup electrodes	Subjects with ↑ temporal delta source activation more likely to progress to dementia at ~ 14 months later
Nobili et al., 1999 [58]	Probable AD (*n* = 72)	16 scalp electrodes but only 2 channels for each hemisphere selected	Loss of ADLs predicted by delta power; incontinence predicted by right-sided alpha power
Moretti, 2015 [59]	MCI (*n* = 74)	19 electrodes referenced to both mastoids	↑ Ratios of high/low EEG alpha power were associated with more cortical atrophy, ↓ temporo-parietal perfusion, and a greater probability of developing dementia at 3-year follow-up

*AD* Alzheimer’s disease, *Ag* silver, *AgCl* silver chloride, *EEG* electroencephalogram, *GFP* global field power, *MCI* mild cognitive impairment, *SCD* subjective cognitive decline

**Table 2:** Awake or resting-state EEG of cognitive vulnerability to ADRD

Section	Ref no	Study type	Sample size	Patient state	EEG system	No. of EEG electrodes	Main result(s)	Data quality*
Awake or resting-state EEG of cognitive vulnerability to ADRD	[13]	RCT	92	Perioperative	BIS (Aspect Medical Systems)	4	↓BIS values at baseline, during loss of consciousness, after discontinuing anesthesia, and before extubation in patients with cognitive impairment	II
	[25]	PC	79 healthy elderly, 53 SMC, 51 non-amnesic MCI, 92 amnesic MCI	Resting state	Not specified—standard 10–20 locations	19	Several differences in the magnitude and topography of source power in different frequencies between groups (healthy elderly, SMC, non-amnesic MCI, amnesic MCI)	III
	[27]	RC	30 healthy elderly, 30 MCI due to AD, 23 LBD	Resting state	Not specified—standard 10–20 locations	19	Both MCI groups showed slowing of individual alpha frequency peak, abnormal lower posterior alpha source activities, and higher widespread delta source activities	III
	[28]	PC	125 AD, 60 controls	Resting state	Neuroscan Medical Systems (Neurosoft Inc. Sterling, VA)	19	AD patients showed patterns of functional network disruption in theta and alpha bands	II
	[30]	PC	84 MCI, 65 AD	Resting state	Not specified—standard 10–20 locations	19	Carriers of the late-onset AD risk factor CST3 haplotype B showed ↓ amplitudes of alpha 1 and alpha 2 than non-carriers did	II
	[31]	PC	89 MCI, 103 AD	Resting state	Not specified—standard 10–20 locations	19	ApoE4 carriers showed ↓ amplitudes of alpha 1 and 2 sources than non-carriers did	II
	[33]	PC	69 healthy elderly, 88 MCI, 109 mild AD	Resting state	Not specified—standard 10–20 locations	19	Frontoparietal delta and alpha synchronization likelihood ↓ progressively through MCI and AD	II
	[34]	RC	30 healthy elderly, 30 with MCI due to AD, 23 with MCI due to LBD	Resting state	Not specified—standard 10–20 locations	19	Patients with MCI due to AD or LBD have ↓ lagged linear connectivity in alpha sources than controls do	III
	[35]	PC	40 healthy elderly, 78 amnesic MCI	Resting state	Not specified—standard 10–20 locations	19	↓ frontoparietal coupling in MCI patients than in controls	II
	[36]	RC	28 healthy elderly, 57 MCI	Resting state	Not specified—standard 10–20 locations	19	↓ total coherence of alpha 1 rhythms in MCI patients than in controls, and negatively correlated with cholinergic damage	III
	[37]	PC	210 SCD, 230 MCI, 197 AD	Resting state	Not specified—standard 10–20 locations	19	↓ CSF Aβ42 was correlated with ↑ theta and delta GFP; ↑ p- and t-tau levels were correlated with ↓ alpha and beta GFP; both ↓ Aβ42 and ↑ p- and t-tau were associated with ↓ global field synchronization	II
	[38]	PC	318 SMC	Resting state	Electrical Geodesics, Inc. (Eugene, OR)	256	There was no strong effect of amyloid load on functional connectivity	II
	[39]	PC	318 SMC	Resting state	Electrical Geodesics, Inc. (Eugene, OR)	256	Elderly patients with higher education (bachelor’s and up) exhibit changes in posterior alpha rhythms	II

*AD* Alzheimer’s disease, *BIS* Bispectral Index, *CSF* cerebrospinal fluid, *GFP* global field power, *LBD* Lewy body dementia, *MCI* mild cognitive impairment, *PC* prospective cohort study, *RC* retrospective cohort study, *RCT* randomized controlled trial, *SMC* subjective memory complaints

*Data quality was assessed per guidelines provided in https://www.ncbi.nlm.nih.gov/pmc/articles/PMC3838756/

## Preoperative EEG and Delirium

Delirium seems to be the result of an altered external information processing, due to a derangement of networks in which acetylcholine is a key modulator, especially in regulating the activity of the thalamus together with cortical areas [60]. In addition to cholinergic impairment, the thalamus plays a pivotal role in the pathogenesis of disorders of consciousness, and its derangement is considered the driver for the generation of thalamocortical dysfunction (TCD). The TCD theory postulates that high-order thalamic nuclei, whose activity is modulated by cholinergic neurons that project from the pedunculopontine nuclei (PPN) to the reticular thalamic nuclei (TNR), enter into a theta burst-firing mode that drives dysfunctional cortical activity.

From a neurophysiological point of view, TCD is characterized by the progressive appearance of a pre-alpha or theta rhythm on EEG. This EEG rhythm is also considered a main hallmark of LBD, has been associated with fluctuations of consciousness even in the prodromal stages of the disease, and has been reported as a supportive biomarker for the diagnosis of LBD [60]. Therefore, considering that a disturbance of consciousness is an essential prerequisite for diagnosing delirium, and taking into account the role of thalamocortical system in consciousness, attention and awareness, TCD (unveiled by the appearance of pre-alpha rhythm) could play a role in delirium pathophysiology and could be an underpinning of prodromal LBD-related delirium.

Furthermore, aside from this role in identifying patients at risk for ADRD (and who merit intervention), awake preoperative EEG abnormalities have been identified in individuals who later developed postoperative delirium. For example, preoperative EEG slowing has been associated with subsequent postoperative delirium [61].

Additionally, predisposing factors that have been associated with increased risk for delirium (e.g., older age, cognitive impairment, alcohol misuse, physical status and functional impairment [62]) have been associated with decreased alpha connectivity strength [63] between EEG electrodes in older adults without delirium. Finally, a recent pilot study found that the magnitude of preoperative alpha attenuation at posterior EEG electrodes (i.e., the reduction in alpha power when older adults go from the eyes-closed, awake state to the eyes-open, awake state) was associated with the severity of postoperative delirium [64].

Similar to the state of EEG changes in patients with SMC, MCI, and AD, preoperative EEG alterations have been demonstrated in patients who later developed postoperative delirium. In both cases, while these alterations may not have sufficient sensitivity and specificity to predict exactly which patients would develop a disorder (i.e., either ADRD or postoperative delirium), perioperative EEG changes could nonetheless be used to identify patients at higher risk who could then be selected for further workup. In the case of postoperative delirium, this is particularly important because there are behavioral interventions that can reduce delirium risk (such as the HELP program [65] and the ABCDEF bundle [66]), but these interventions are often too resource-intensive to apply universally to all older surgical patients. Thus, preoperative EEG could be used as an additional screening test to identify which older patients are at increased risk for postoperative delirium, and thus who might benefit most from the HELP or ABCDEF interventions.

## Traditional Uses for Intraoperative EEG—Preventing Awareness with Explicit Recall and Titrating Anesthetics

Intraoperative EEG monitoring (typically with 2–4 frontal electrode arrays) are used at many medical centers for anesthetic titration. EEG can be used for anesthetic titration because anesthetic drugs with GABA-A agonist activity (such as propofol and the halogenated volatile agents: isoflurane, sevoflurane and desflurane), produce characteristic EEG waveform changes, i.e., delta (< 4 Hz) and alpha (8–12 Hz) oscillations at frontal scalp sites, with the halogenated volatile agents also producing theta oscillations. In contrast, drugs with other mechanisms of action such as alpha-2 adrenergic receptor agonists (i.e., dexmedetomidine) or NMDA (N-methyl-D-aspartate) antagonists (i.e., nitrous oxide, ketamine, and xenon [67]) tend to produce other characteristic EEG waveform patterns (reviewed in [68]).

With increasing dosage of inhaled anesthetics and propofol, frontal EEG waveforms progress through a period of paradoxical excitation marked by increased beta oscillation power (12–30 Hz) before slow-delta (< 4 Hz) and alpha oscillation power (8–12 Hz) become dominant and beta and gamma (> 30 Hz) oscillation power diminishes significantly. This pattern of high amplitude slow-delta and alpha oscillations in ongoing EEG activity is thought to be associated with both a low incidence of patient movement in response to noxious stimuli and a low incidence of intraoperative awareness with explicit recall [68–71]. This behavioral state is generally considered the desired end point for general anesthesia. Further increases in inhaled anesthetic dosage then lead to decreases in frontal alpha power [72], followed by the emergence of burst suppression—an intermittent isoelectric (flat line) EEG signal between bursts of delta and/or alpha oscillations [73].

Several commercial EEG monitors also produce processed index values, typically on a scale of 0 to 100, in which awake conscious individuals typically have values in the 90–100 range. The manufacturers of several of these devices (most notably the BIS, or Bispectral Index monitor [74]) have marketed them with the claim that using these processed EEG monitors (and titrating anesthetic drug delivery to maintain the processed EEG within the target range) would help reduce intraoperative awareness with explicit recall. Despite this claim, the evidence supporting the use of processed EEG monitors for the prevention of intraoperative awareness is inconsistent, and may depend on the patient population in which the monitor is utilized. There is evidence that use of the BIS monitor in patients at high risk of awareness [75] and receiving total intravenous anesthesia (TIVA) [76] might reduce the incidence of intraoperative awareness. However, there appears to be no additional benefit provided by BIS monitoring in patients receiving inhalational anesthesia with continuous monitoring of end-tidal gas concentrations [77]. Overall, the consensus in the field is that intraoperative EEG monitoring may help reduce intraoperative awareness rates when used in TIVA cases, but not for cases performed with volatile anesthetics with end-tidal gas monitoring [78].

Interestingly, there is also evidence to suggest that the use of processed EEG monitors—such as Narcotrend (Narcotrend^®^; MonitorTechnik, Bad Bramstedt, Germany), the BIS monitor (BIS^®^; Aspect Medical Systems, Newton, MA), or Sedline (Masimo Corporation, Irvine, CA)—that provide measures of a patient’s level of consciousness may help speed up emergence from anesthesia. Studies have found that use of these monitors results in shorter time to eye opening, faster extubation, higher orientation upon arrival to the recovery room/area, and earlier qualification for discharge from the anesthesia recovery room/area [79–82]. Although trials have examined whether intraoperative BIS monitor use reduces delirium risk (discussed below in the section titled *EEG Monitoring for Reducing Postoperative Delirium Risk*), observational studies have generally not observed different intraoperative BIS values among patients with vs. without delirium [83, 84] (see Table 3 for data quality ratings of observational intraoperative EEG studies discussed here).

**Table 3:** Observational intraoperative EEG studies

Section	Ref no	Study type	Sample size	Patient state	EEG system	No. of EEG electrodes	Main result(s)	Data quality
Observational intraoperative EEG studies	[61]	PC	237	Perioperative	Sedline (Masimo Corporation, Irvine, CA)	4	↓ Preoperative SEF, absence of change in SEF during loss of consciousness, and ↓ intraoperative alpha power are related to development of POD	II
	[85]	PC	35 BIS, 15 32-channel	Perioperative	BIS (Medtronic/Covidien, Dublin, Ireland), BrainAmp MR Plus (Brain Products GmbH, Gilching, Germany)	4 or 32	↓ Intraoperative alpha power was associated with preoperative cognitive impairment	III
	[83]	PC	139	Intraoperative	BIS (Covidien, Dublin, Ireland)	4	Intraoperative BIS did not differ between patients with vs. without POD; ↓ EEG measure of anesthetic resistance (DARS) was associated with ↑ POD risk	II
	[87]	PC	38	Perioperative	Sedline (Masimo Corporation, Irvine, CA)	4	↓ Intraoperative alpha power was associated with preoperative cognitive impairment	III
	[88]	PC	30	Intraoperative	Biosemi (Amsterdam, Netherlands)	16	↓ Intraoperative alpha power was associated with changes in delirium status as identified by CAM assessments	III
	[89]	RC	618	Intraoperative	BIS (Covidien, Dublin, Ireland)	4	Burst suppression at ↓ anesthetic concentrations (i.e., increased anesthetic sensitivity) was associated with ↑ POD risk	II
	[91]	PC	626	Intraoperative	Sedline (Masimo Corporation, Irvine, CA, USA), BIS (Covidien, Dublin, Ireland)	4	Burst suppression and EEG emergence trajectories with ↓ spindle power were associated with PACU delirium	II
	[92]	PC	41	Intraoperative	Sedline (Masimo Corporation, Irvine, CA)	4	Burst suppression as measured by visual analysis was associated with POD	III
	[93]	PC	727	Intraoperative	BIS (Covidien, Dublin, Ireland)	4	Patients with EEG suppression were more likely to experience POD	II
	[94]	PC	81	Perioperative	BIS (Unspecified)	4	Patients with POD experienced ↑ durations of intraoperative burst suppression	III
	[97]	PC	50	Perioperative	BrainAmp MR Plus (Brain Products GmbH, Gilching, Germany), LiveAmp (Brain Products GmbH, Morrisville, NC)	32	The scale at which pre- and intraoperative EEG complexity are equal may be predictive of delirium	III
	[98]	PC	89	Preoperative, Postoperative	Electrical Geodesics, Inc. (Eugene, OR)	256	EEG complexity is negatively correlated with delirium severity	III
	[96]	RC	141	Intraoperative	Sedline (Masimo Corporation, Irvine, CA)	4	↑ Delirium incidence in those with vs without EEG burst suppression (BSup); BSup mediated EEG alpha power, physical function, and lowest temperature during bypass on delirium; delirium associated with ↓ 6.8–24.4 Hz power	II

*CAM* confusion assessment method, *DARS* Duke Anesthetic Resistance Scale, *PACU* post-anesthesia care unit, *PC* prospective cohort study, *POD* postoperative delirium, *RC* retrospective cohort study

## Intraoperative EEG Monitoring to Identify Patients at Increased Delirium Risk

Aside from their original proposed role in reducing the incidence of intraoperative awareness (and for accelerating emergence from general anesthesia), there are two related intraoperative EEG features that have been associated with postoperative delirium: decreased alpha power and increased burst suppression. Decreased intraoperative alpha power (i.e., in response to inhaled anesthetics or propofol) has been shown to correlate with preoperative cognitive impairment [85], which is a known predisposing factor for postoperative delirium [86]. Subsequent work corroborated this finding [87], and went on to show that decreased intraoperative alpha power was associated with the severity of postoperative sub-syndromal delirium [88]. Separately, several studies found that increased incidence and duration of intraoperative burst suppression (i.e., intermittent periods of a flat line EEG signal) were associated with increased delirium risk [89–94]. Interestingly, subsequent work has shown that increased incidence and duration of burst suppression were also associated with decreased intraoperative EEG alpha power [95, 96].

Aside from specific EEG features such as alpha power and burst suppression, EEG complexity, a measure of temporal fluctuations or irregularities in the EEG signal, has also been studied with respect to delirium. Acker and colleagues found that overall EEG complexity, quantified by multi-scale entropy (MSE), was not associated with delirium. However, they found a “crossover” point where the preoperative and intraoperative MSE curves intersected. This crossover point represented the time scale at which the preoperative and intraoperative complexity or entropy of the EEG signal was equal. Interestingly, when correlating preoperative to intraoperative MSE crossover point with delirium severity, individuals with crossover points at shorter time scales trended toward greater delirium symptomatology [97]. Similarly, reduced EEG complexity as measured by Lempel–Ziv Complexity (considered to represent a proxy for cortical information content) has also been correlated with delirium in a scalp-wide fashion [98].

In contrast to these clear-cut associations between complexity measures (and other intraoperative EEG features) with postoperative delirium, as noted above, observational and retrospective studies have generally not found an association between intraoperative BIS index values and postoperative delirium. This is surprising, partly because the BIS index algorithm itself partly relies on burst suppression measurements [99]. A potential explanation may lie in the principle that BIS index values, like the processed EEG features described above, reflect both the preoperative structure and function of the patient’s brain as well as its responses to surgical stimuli and varying anesthetic drug dosage [100]. To effectively control for anesthetic dosage effects on BIS values and isolate the effect of preoperative neurocognitive impairment on these BIS values, a recent report studied the effect of dividing case average BIS values by the difference between the maximum inhaled anesthetic dose likely to be given in clinical practice (2.5 aaMAC, where aaMAC is the age-adjusted end-tidal minimum alveolar concentration) and the actual mean inhaled anesthetic dose received by a given patient [83]. Low values on this anesthetic resistance scale, calculated by [BIS/(2.5-aaMAC)], imply that a patient has lower BIS values than would be expected for a given inhaled anesthetic dose (see Box 1). Low values on this anesthetic resistance scale were highly associated with postoperative delirium incidence in a combined cohort of older patients from two different institutions [83]. Interestingly, low values on this anesthetic resistance scale [BIS/(2.5-aaMAC)] were independently associated with delirium risk in multivariable adjusted analyses, even though neither BIS nor aaMAC themselves were associated with delirium risk.

Similarly, Fritz and colleagues found that increased intraoperative anesthetic sensitivity (i.e., an increased propensity for burst suppression at low anesthetic doses; see Box 1) was highly associated with postoperative delirium, while burst suppression alone (i.e., without adjustment for anesthetic dose [89]) was not associated with postoperative delirium in this study. Taken together, these two studies suggest that both processed and raw EEG features adjusted for the anesthetic dose received by individual patients may be more closely associated with postoperative delirium than the unadjusted versions of these indices, thus emphasizing the importance of using “anesthetic-adjusted” EEG indices in future studies relating intraoperative EEG parameters and postoperative delirium. Further, there is reason to think that studying preoperative “unprovoked” resting-state EEG activity together with EEG responses to anesthetic administration may be more indicative of brain structure and function among older adults than either type of EEG recording alone, since a general principle of geriatrics is that physiologic responses to a stressor (i.e., provocative tests) provide an informative test of functional capacity (or reserve) within relevant organ systems [101].

Box 1 Select anesthetic-adjusted EEG measures

**Table:** 

**Citation**	**Anesthetic-adjusted EEG measure equation**
(Cooter Wright et al., 2022) [83]	$DARS=BIS \left(\frac{1}{2.5-aaMAC}\right)$
(Fritz et al., 2018) [89]	$logit{\left(SR>0\right)}_{i,t}= \frac{1}{1+{e}^{-({\beta }_{0 }+ {u}_{0i }+ {\beta }_{1}{\left(ETAC\right)}_{i,t} + {\beta }_{2}{(Propofol\; Dose)}_{i,t }+ {\beta }_{3}{(Midazolam\; Dose)}_{i }+ {\beta }_{4}{(Opioid\; Dose)}_{i }+ {\beta }_{5}{(Nitrous\; Oxide)}_{i})}}$

*DARS* Duke Anesthesia Resistance Scale, *BIS* Bispectral Index, *aaMAC* age-adjusted end-tidal minimum alveolar concentration, *logit* a log-odds function used to represent probability as a value from 0 to 1 in logistic regression models, *SR* suppression ratio, *ETAC* end-tidal anesthetic concentration

## EEG Monitoring for Reducing Postoperative Delirium Risk

The associations between delirium and processed EEG values that potentially indicate “excessive” anesthetic dosage have led to a number of studies that have attempted to reduce postoperative delirium incidence by titrating anesthetic administration in response to these EEG parameters. These studies have generally assessed delirium rates (and sometimes delirium severity) after randomizing patients to either: (1) BIS-guided vs. non-BIS-guided anesthetic titration [102, 103]; (2) two different target BIS ranges (i.e., deep vs. light anesthesia or sedation groups [104, 105]); (3) usual care versus an anesthetic regimen designed to reduce the incidence of both burst suppression and low BIS values [106]; or (4) general anesthesia without EEG guidance vs BIS-guided sedation and spinal anesthesia [107]. Overall, a recent meta-analysis found no effect of processed EEG-guided anesthetic administration on postoperative delirium rates across nine studies, although a positive effect was seen in a pre-planned secondary analysis using a random effects model [108]. Yet, the authors of this meta-analysis concluded their meta-analysis abstract by noting that, related particularly to the four study models listed above, “high clinical heterogeneity limits the inferences from this and any future meta-analyses” [108] on the effect of processed EEG-based anesthetic titration on postoperative delirium risk. To date, no published studies have examined the effect of anesthetic titration based on EEG alpha power on postoperative delirium risk, although one randomized trial found that anesthetic titration based on maximizing frontal alpha power led to decreased hospital length of stay (an exploratory outcome in this study [109]). An additional study comparing the effect of anesthetic titration to maximize EEG alpha power vs usual care on delirium outcomes is currently underway, with results expected in 2023 [110, 111].

One reason for the high heterogeneity mentioned above across the studies using processed EEG monitoring to reduce postoperative delirium rates is that clinicians were directed to use EEG parameters in different ways across these various studies to alter anesthetic management [102–107] (Table 4). Perhaps as a result, clinicians altered anesthetic dosage to varying extents across these studies in response to information from the EEG monitors [102–107]. Thus, as with any investigation of the effect of a new or additional intraoperative monitor on patient outcomes, the divergent outcomes across these studies may be a reflection of how different clinicians used the information from these EEG monitors to alter care in different ways, rather than an indication of the effect of EEG monitoring itself on patient outcomes [112].

**Table 4:** Intraoperative EEG for reducing postoperative delirium and/or improving other outcomes, as well as studies on EEG and emergence

Section	Ref no	Study type	Sample size	Patient state	EEG system	No. of EEG electrodes	Main result(s)	Data quality
Intraoperative EEG for reducing postoperative delirium and/or improving other outcomes	[102]	RCT	921	Intraoperative	BIS (Covidien, Mansfield, MA)	4	Use of BIS ↓ delivery of propofol and volatile anesthetic and ↓ risk of POCD at 3 months and POD	I
	[103]	RCT	1155	Intraoperative	BIS (Covidien, Boulder, CO)	4	BIS-guided anesthesia titration resulted in ↓ delirium incidence	I
	[104]	RCT	114	Intraoperative	BIS (Aspect Medical System Inc, Norwood, MA)	4	The incidence of postoperative delirium was ↓ in the group receiving lighter sedation (BIS ≥ 80) than in the group receiving deep sedation (~ 50)	I
	[105]	RCT	515	Intraoperative	BIS (Unspecified)	4	↓ POD incidence in group receiving lighter sedation (BIS 50) than in the group receiving deep sedation (35); ↑ cognitive function at 1 year in the BIS 50 group than the BIS 35 group	I
	[106]	RCT	1232	Intraoperative	BIS (Medtronic)	4	EEG-guided anesthetic administration did not decrease the incidence of postoperative delirium compared to usual care	I
	[107]	RCT	217	Intraoperative	BIS (Unspecified)	4	Spinal anesthesia with BIS-guided sedation titration did not ↓ delirium relative to general anesthesia with masked BIS	I
	[109]	RCT	484	Intraoperative	Sedline (Masimo Corporation, Irvine, CA)	4	↓ postoperative length of stay in patients cared for by residents undergoing an electronic learning curriculum to titrate anesthetics based on EEG spectrogram interpretation	I
EEG and emergence	[116]	PC	52	Perioperative	Bio-AMP8 (Bio-Acquisition Systems, Kangpu Medical, Huzhou, Zhejiang, China), BIS (Aspect Medical System, Newton, MA)	2 and 4	Four patterns were identified in EEG data during emergence; these patterns are age-related	III
	[117]	PC	60	Perioperative	Electrical Geodesics, Inc. (Eugene, OR)	32, 64, or 128	The peak frequency of the posterior dominant rhythm was initially ↓ at the time of return of responsiveness; afterwards, posterior dominant rhythm peak frequency was associated with reaction time and accuracy during cognitive testing	II
	[118]	PC	8	Perioperative	Electrical Geodesics, Inc. (Eugene, OR)	128	Posterior alpha power was ↓ during unconsciousness and recovered to baseline over 3 h	III
	[119]	PC	53	Postoperative	Cognionics, Inc. (San Diego, CA)	16	Alpha parietal power and frontal-parietal connectivity were the predominant features seen on postoperative EEG in PACU	III
	[121]	PC	20	Perianesthesia	Electrical Geodesics, Inc. (Eugene, OR)	128	EEG networks returned to baseline in 30–60 min after recovery of consciousness	III
	[122]	PC	60	Perianesthesia	Electrical Geodesics, Inc. (Eugene, OR)	32, 64, or 128	Cognitive performance of patients undergoing anesthesia was similar overall within 3 h after return of consciousness to patients that did not undergo anesthesia	III
	[123]	PC	116	Perianesthesia	NIM-Eclipse neuromonitoring system (Medtronic, Dublin, Ireland)	10	Patients with PACU delirium had ↓ relative alpha power and ↓ fronto-parietal alpha coherence	II

*PACU* post-anesthesia care unit, *PC* prospective cohort study, *POD* postoperative delirium, *POCD* postoperative cognitive dysfunction, *RC* retrospective cohort study, *RCT* randomized controlled trial

Various other explanations for these divergent outcomes have also been discussed, including differences in a priori study registration, differences in the ancestral origin of the cohorts in different studies (with resultant differences in drug metabolism [113]), differing effects of altering anesthetic dosage on patients with normal vs. impaired cognition or on patients with differing degrees of comorbidities, and the fact that an intervention (such as EEG monitoring) can only plausibly modulate an outcome (such as delirium) via numerous intermediate steps subject to confounding [114, 115]. Another way to make sense of this literature is to think of patients in three groups: (1) those who are highly *unlikely* to become delirious after surgery regardless of what intraoperative anesthetic regimen is used; (2) those who are highly *likely* to become delirious after surgery regardless of what intraoperative anesthetic regimen is used; and finally (3) those whose postoperative delirium risk is modifiable based on what intraoperative anesthetic regimen is used. In statistical terms, this is analogous to the view that rather than a fixed effect of EEG-guided anesthetic administration on postoperative delirium incidence, there are likely differing (or random) effects of such interventions on different patient groups.

At present, we do not know how to define which patients belong to each of these three groups, since these hypothetical groups are presumably defined by structural and functional characteristics of the CNS that we do not normally measure prior to anesthesia and surgery. Since we do not know how to define which patients belong to each of these three groups, we do not know the relative distribution of patients from each of these three cohorts within the studies discussed above. Thus, studies that demonstrated differences in delirium rates due to EEG guidance may simply have enrolled a greater percentage of patients in group 3, and vice versa, studies that found no benefit of EEG guidance on delirium rates may have enrolled a relatively greater percentage of patients from groups 1 and 2. While this explanation is theoretically possible, to test it we will need to develop a better understanding of the brain characteristics that predispose patients to postoperative delirium, and the means to measure these brain characteristics across future study participants (or even as a means to select at-risk patients for such studies). Ultimately, larger future studies will be required to better understand whether there are specific sub-populations of patients in whom EEG-guided anesthetic titration will lead to lower delirium rates.

## EEG Monitoring During Anesthetic Emergence

While this discussion above has focused on the relationship between summary or mean EEG patterns during anesthesia and postoperative delirium, there is also evidence that specific EEG patterns seen during emergence from anesthesia may also be associated with the return of normal cognitive function and even with the subsequent development of postoperative delirium (Table 4). In one study of 52 patients undergoing sevoflurane anesthesia, 4 types of EEG patterns were identified during emergence, with differences in emergence time dependent on patient age [116]. The ReCCognition study randomized healthy adults to either 3 h of general anesthesia with isoflurane or resting wakefulness, and found that the post-anesthesia return of responsiveness was initially accompanied by a lower than normal posterior EEG dominant frequency and power (i.e., posterior alpha peak frequency and power), both of which gradually returned to baseline over the next three hours [117, 118]. Interestingly, this gradual return to normal of the posterior dominant alpha peak frequency correlated with improving cognitive task performance over the three hours following return of responsiveness, raising the possibility that the postoperative normalization of posterior EEG alpha peak frequency could be used to track the return of cognitive function in older surgical patients. Similarly, another study evaluating 53 adult surgical patients in the PACU found that a shift to parietal alpha power and frontal-parietal alpha band connectivity were the predominant EEG features that appeared during postoperative recovery [119], although neither feature was associated with the time-to-meet PACU discharge criteria or sedation scores upon initial assessment.

EEG has also been used to identify motifs (or patterns) of inter-connections between nodes or regions of networks in the brain, which have shown changes in topological organization associated with general anesthesia-induced unconsciousness [120]. Peak perturbation of these functional brain networks under anesthesia occurs at return of consciousness with a return to baseline within the first hour, although deficits in cognitive performance can last up to 3 h afterward [121]. EEG return to baseline of frontal-parietal network dynamics as measured by permutation entropy (a local measure of EEG signal fluctuations) and Lempel–Ziv Complexity (a global measure of EEG signal fluctuations) has also been seen at the point of return to consciousness [122]. Taken together, these changes in EEG power, dominant frequency, motifs, and complexity that accompany emergence from anesthesia provide multiple different electrophysiologic biomarkers that could be studied as potential predictors of postoperative delirium or other types of perioperative neurocognitive disorders. One such study of EEG emergence patterns and delirium found that patients who emerged from anesthesia without significant frontal alpha power had an increased risk of developing post-anesthesia care unit (PACU) delirium [90]. Similarly, another report found that as compared to patients without PACU delirium, those with PACU delirium showed lower frontal EEG alpha power and lower frontal-parietal theta and alpha coherence during anesthetic emergence [123]. These EEG emergence patterns were not highly sensitive and specific for the later development of delirium, though they had better predictive ability than age for the development of delirium. Thus, while not diagnostic, these EEG patterns could potentially be used as a rough screening test to gauge relative delirium risk and to select patients for resource-intensive delirium prevention interventions, which may not be available for all patients.

## Postoperative EEG Waveforms and Delirium

EEG recording during delirium has typically showed generalized slowing, characterized by increased power in delta and theta bands with a concomitant decrease in alpha band power [124–130] (Table 5). One study that examined the differences of EEG between patients with and without delirium after undergoing cardiothoracic surgery identified relative delta power across a frontal-parietal electrode derivation as a potential measure to distinguish the two groups of patients, with the largest difference noted during eyes-closed periods [131]. Another study in cardiac surgery patients in the intensive care unit found that median BIS values on postoperative day 1 were significantly lower in delirious patients, with the delirious patients also displaying lower relative alpha and higher theta power [128]. A post-cardiac surgery study compared patients with hypoactive delirium (*n* = 18), patients recovering from anesthesia (*n* = 20), and non-delirious controls (*n* = 20), and found that delirious patients showed less integrated networks in the alpha band than non-delirious controls [127]. The same group examined 159 patients 60 years of age or older undergoing EEG on postoperative days 1 to 3 and found that relative EEG delta power could be used to help detect delirium in these patients [132]. Reduced functional interdependence of EEG scalp electrode pairs has been associated with delirium [126, 133, 134], which supports the hypothesis of delirium as a disorder of disintegrated brain network connectivity [135–137], a finding that has also been supported by resting-state functional MRI [125].

**Table 5:** Postoperative EEG and delirium

Section	Ref no	Study type	Sample size	Patient state	EEG system	No. of EEG electrodes	Main result(s)	Data quality
Postoperative EEG and delirium	[124]	Case report	1	Hospitalization (w/ delirium) and 6 month follow-up	Not specified—standard 10–20 locations	19	The described patient during active delirium had a dominant pattern typical of early-stage LBD	IV
	[126]	Case–control	129 delirium, 414 control	Various, inpatient	Galileo.NET, BE Light system, EB Neuro S.p.A., Firenze, Italy	19	Delirious patients demonstrated global alpha and regional beta band disconnectivity and theta band hyperconnectivity	III
	[127]	PC	18 hypoactive delirium, 20 non-delirium, 20 recovering from anesthesia	Postoperative	Micromed (Trevisio, Italy)	21	Patients recovering from anesthesia or with hypoactive delirium demonstrate ↓ functional and directed EEG connectivity (a less integrated brain network in the alpha frequency band) compared to non-delirious controls	III
	[128]	PC	114	Postoperative / ICU	BIS (Aspect Medical System Inc, Norwood, MA)	4	Patients with delirium had ↓ BIS values and different alpha and theta values on postoperative day 1	II
	[129]	RC	26 delirium, 28 non-delirium	Postoperative	Micromed (Trevisio, Italy)	21	Patients with delirium had ↑ spectral variability (determined by the coefficient of variation) in alpha and beta, ↓ spectral variability in delta, and ↓ complexity relative to patients without delirium	III
	[130]	PC	26	Postoperative	Biopac MP150, Bionomadix BN-EEG2 wireless modules	3	Eyes-closed occipital alpha relative power varied inversely with delirium severity; eyes-open occipital theta relative power was correlated with severity of delirium	III
	[132]	PC	159	Perioperative	Not specified—standard 10–20 locations	3	Relative delta EEG power in one channel was correlated with clinically diagnosed delirium	II
	[133]	PC	70	Perioperative	Electrical Geodesics, Inc. (Eugene, OR)	256	Delirium was associated with ↑ slow-wave activity in posterior regions and ↓ functional connectivity	III
	[134]	Case–control	49	Postoperative	Micromed (Trevisio, Italy)	21	Delirium was associated with ↓ alpha band functional connectivity and ↑ delta band connectivity	III
	[138]	PC	200	Hospitalization	Micromed (Trevisio, Italy)	21	Generalized slowing in theta and delta were associated with delirium severity	II
	[139]	PC	54	Postoperative	Electrical Geodesics, Inc. (Eugene, OR)	256	Postoperative EEG delta power was associated with delirium severity	II
	[140]	PC	3 delirium, 9 non-delirium	Postoperative	Philips Alice PDx portable sleep systems (Philips-Respironics, Murrysville, PA)	Up to 28	Delta power on postoperative day 1 (↑ waking power and ↓ non-REM sleep power) was associated with postoperative day 2 delirium severity	III

*LBD* Lewy body dementia, *PC* prospective cohort study, *RC* retrospective cohort study

In addition to these correlations with delirium incidence, postoperative awake resting-state EEG features such as generalized slowing in frequency have also been found to correlate with delirium severity and poor clinical outcomes [138]. Similarly, increased postoperative resting-state EEG delta power has been associated with both increased delirium severity and (paradoxically) with increased preoperative cortical thickness [139]. Another study of 12 patients undergoing orthopedic surgery found that greater resting-state delta power while awake on postoperative day 1, and lower EEG delta power in non-REM sleep on night 2, predicted the severity of delirium on postoperative day 2 [140]. Reduced complexity of the resting-state EEG signal, which may reflect fewer and/or simpler patterns of neural activity within the brain and lower cortical information content (as mentioned earlier in this review), has also been correlated with delirium severity [98]. A major question for the field is whether EEG correlates of delirium reflect underlying vulnerabilities that indicate delirium risk versus whether they measure the brain activity patterns of delirium itself.

## EEG When Delirium Is Superimposed on Dementia

A challenging clinical picture emerges when delirium is superimposed on dementia, since some signs and symptoms overlap between these disorders. Thus, further refinement of electrophysiological activity during an episode of delirium, as distinct from pathological EEG findings associated with dementia, may aid the diagnosis of delirium when superimposed on dementia [141–143]. To date, EEG has not been able to differentiate between these two states [144]. The fluctuating arousal and inattentive nature of delirium overlap with features of LBD both phenotypically and electrophysiologically [124]. In a recent study, patients with delirium exhibited higher dominant frequency (DF) variability in the pre-alpha/theta range (6–7.5 Hz), in addition to an overall lower dominant frequency, a pattern resembling that observed in LBD [60]. These similarities potentially reflect vulnerability in brainstem networks which govern the transition between, and maintenance of, different cortical states. The identification of the aforementioned EEG activity patterns before or after surgery, which resemble those seen in LBD, provides an opportunity to select patients for alpha-synuclein CSF level measurements (a LBD biomarker) and further workup for LBD [145, 146]. Similarly, since patients with postoperative delirium are more likely to have preclinical amyloid or tau pathology (as indicated by low CSF amyloid and high CSF tau levels [147, 148]), the development of postoperative delirium (or EEG patterns associated with it) could be used to identify patients at risk for developing AD for clinical trials and other interventions (such as intensive blood pressure management) that have been shown to reduce MCI and dementia risk [149].

The aberrant EEG activity discussed above that is seen during delirium (Table 6) is thought to reflect thalamocortical dysrhythmia, and normalizes following delirium resolution in most patients [60]. This not only sheds light on potential systems neuroscience-level mechanisms of delirium, but also may be useful when assessing delirium in people who are hypoactive or even catatonic and thus may not be able to complete delirium screening assessments. Furthermore, if the transient neurophysiological disruption seen during delirium could be further differentiated from aberrant EEG activity patterns seen in people with dementia, then these delirium-specific EEG patterns (in addition to other measures) could be a useful tool for the assessment of delirium superimposed on dementia.

**Table 6:** EEG for delirium and dementia, after delirium resolution, and in the ICU

Section	Ref no	Study type	Sample size	Patient state	EEG system	No. of EEG electrodes	Main result(s)	Data quality
EEG for delirium and dementia	[144]	PC	18 delirium, 43 delirium-demented	Hospitalization	Not specified	Not specified	EEG was not able to differentiate patients with delirium vs. patients with both delirium and dementia	III
	[60]	PC	65 delirium, 41 non-delirium	Resting state	Not specified—standard 10–20 locations	19	Patients with delirium had ↑ dominant frequency variability in prealpha/theta frequencies and ↓ dominant frequency overall, similar to patients with LBD; these abnormalities normalized by discharge from hospital or 1-month follow up	II
EEG after delirium resolution	[151]	PC	28	Resting state	Neurotrac (Interspec, Inc.)	2	Persistent EEG slowing was seen at four months after hospital discharge	III
	[152]	PC	96	Resting state	Neurotrac (Interspec, Inc.)	2	Persistent EEG slowing was seen at one year after hospital discharge	III
EEG in the ICU	[154]	Post hoc analysis of cohort	125	ICU (ventilated)	BIS (Aspect Medical System Inc, Norwood, MA)	3 or 4	Burst suppression was associated with ↑ risk of mortality at 6 months	III
	[155]	Post hoc analysis of cohort	10	Hospitalization	Nicolet and NicoletOne EEG Reader™ (Natus Medical Inc., Pleasanton, CA)	19	Generalized EEG background slowing during hospitalization; cognitive impairment in ≥ 1 domain in all patients at 12-month follow-up, with some EEG feature correlates—relative alpha power with visuospatial/constructional, and peak interhemispheric coherence negatively with delayed memory	III
	[84]	PC	124	ICU (ventilated)	BIS (Covidien)	4	After controlling for arousal levels, BIS algorithms (XP, 3.4) did not distinguish between presence vs. absence of delirium	II
	[94]	PC	69	ICU (ventilated)	BIS (Aspect Medical Systems)	4	Duration of burst suppression during coma was associated with prevalence and duration of delirium	III

*ICU* intensive care unit, *LBD* Lewy body dementia, *PC* prospective cohort study

## EEG Recorded Following Delirium Resolution

There is a dearth of studies that have recorded resting-state EEG longitudinally in people whose delirium has resolved [60, 150]. Persistent resting-state EEG slowing in frequency was seen at four months [151] and one year [152] following hospital discharge among older adults in two studies, while one study found an overall normalization of quantitative resting-state EEG patterns at one month after discharge in a majority of people who had delirium [60] (Table 6). Further longitudinal studies are required to determine if delirium is associated with long-term EEG changes, and additional studies of this type are currently underway [153].

## Use of EEG in Intensive Care Units

The use of EEG in other areas such as intensive care units (ICUs) may also provide useful insights for identifying delirium risk, and into the underlying structural and functional brain pathology that may predispose patients to longer-term dementia risk. The existing literature has primarily investigated this in medical ICUs, but these studies still provide relevant insight into how EEG may be used in surgical ICUs.

In one group of sedated, critically ill patients, 39% showed EEG burst suppression, defined as a non-zero burst suppression ratio as calculated by the standard BIS A1050 algorithm. When mortality was assessed 6 months later, burst suppression was an independent predictor of increased mortality risk [154]. In a cohort of 124 mechanically ventilated ICU patients, 69 emerged from coma, of which 42 (61%) met criteria for post-coma delirium. In evaluating EEG patterns associated with coma, most patients were found to exhibit burst suppression, even if short in duration [94]. In a group of 10 ICU survivors, 9 of 10 patients had inpatient EEGs that showed generalized background slowing and 10 of 10 patients demonstrated cognitive impairment in one or more domains at 12-month follow-up. The relative alpha power of the inpatient EEGs was correlated with score on visuospatial/constructional domain tasks, and peak interhemispheric coherence correlated negatively with delayed memory [155]. A systematic review of 14 studies that investigated ongoing EEG characteristics defining delirium in the ICU also found that a reduction in relative alpha power and an increase in relative delta and theta power often distinguished delirium patients from non-delirium patients [156]. Other EEG features such as network topology of the interdependence of EEG activity at electrode pairs and phase lag index, a commonly used, de-biased measure of functional connectivity strength, provide additional insight into neurobiological underpinnings of delirium [134]. In summary, ongoing EEG features such as burst suppression, relative delta through alpha power, and network topology have associations with delirium in patients admitted to medical ICUs (Table 6), suggesting that it is worth investigating the relationship between these EEG patterns and delirium in surgical ICU patients as well.

## Acquiring Further Clinical Workup

An atypical preoperative, intraoperative, or postoperative EEG profile suggestive of either (1) delirium risk, (2) brain abnormalities, and/or (3) unusual response patterns to anesthesia provides an opportunity to modify care planning and decision making. In addition to altering anesthesia management, perioperative EEG profiles could help target at-risk patients for optimization and intervention programs. Atypical EEG profiles could result in referral to geriatricians, behavioral neurologists, and/or neuropsychologists for differential diagnosis and long-term care management. These data would additionally provide objective information to assist in differentiating dementia from postoperative delirium [157]; distinguishing delirium from dementia has been particularly challenging in a postoperative setting particularly if the patient has no caregiver, no medical history, no dementia diagnosis, or cognitive screening recorded [62]. With additional research justifying inclusion into the care pathway, perioperative EEG methods could become a logical expansion for programs such as the American College of Surgeons Geriatric Surgery Verification (ACS GSV) Program, which is designed to improve perioperative care for older adults undergoing surgery [158].

## Practical Barriers to Implementation of Perioperative EEG

Although we lack exact nationwide or worldwide statistics on how often intraoperative EEG monitoring is used, intraoperative EEG is used in up to 1/3 of cases at one of our centers (i.e., Duke Medical Center) [159]. The use of intraoperative EEG requires an understanding of how to (1) correctly and efficiently apply electrodes, (2) establish a high-quality signal, (3) monitor the data for quality and limit artifact in real time, (4) disinfect the equipment after recording (if it is to be reused), and (5) pre-process and analyze the data if that is the goal; this last step is not required if using the EEG solely during surgery (such as for anesthetic titration). We provide a general overview of training requirements below.

1. It is critical that researchers and staff are trained on correctly placing electrodes on the scalp as intended by the manufacturer (i.e., at the x, y, z coordinates specified by the EEG montage, or electrode layout, of the EEG system). These manufacturer-provided electrode locations are used during data processing and analysis; thus, deviations from these coordinates can limit the quality of the data and conclusions. For instance, a multi-channel EEG cap should not be askew to the left or right; electrodes should be within 1 cm of their correct locations (e.g., Cz should be within 1 cm of the center of the scalp).
2. Once electrodes are placed, the gap between the electrodes and the scalp is bridged with a conductive solution (e.g., saline or gel, if the EEG system uses wet electrodes). This conductive solution should be carefully but thoroughly applied to reduce high impedances (i.e., impediments to the flow of the electrical current) while avoiding bridged signals between electrodes that would reduce spatial accuracy [160]. During intraoperative recordings in which the patient may be repositioned for surgery, it is important that both the electrode locations and signal quality remain consistent if using the full intraoperative recording as a single dataset.
3. Data quality should be monitored, with attention to proper electrode locations and signal quality requirements, including the appearance of artifact in the signal (e.g., blinks and eye movement, muscle activity, line noise, environmental noise) and methods should be used to minimize the impact of these issues on the data, such as by isolating the equipment to avoid external electrical interference.
4. After recording is complete, it is important to use proper disinfecting procedures if the recording apparatus is to be reused. Most, if not all, EEG system manufacturers have recommended guidelines for disinfecting the equipment without damaging them; researchers and staff should also be trained on how to disinfect surrounding equipment (e.g., conductive solution applicators, tables, laptops, etc.).
5. After all recording procedures are complete, EEG data are pre-processed to remove noise and retain signals from brain activity. This requires training on signal processing techniques such as filtering, down-sampling, re-referencing, electrode interpolation, and removal of remaining artifacts [161, 162].
6. Subsequent analysis of the pre-processed EEG data generally requires a theoretical understanding of (i) regions of interest; (ii) EEG features (e.g., alpha power, functional connectivity metrics, etc.); and (iii) associated quantitative techniques (e.g., event-related potential analysis, power analysis, etc.) to optimize the EEG analysis for the research question and study design. The potential research insights as well as advantages and disadvantages of different EEG systems are provided in Table 7.

**Table 7:** Advantages, disadvantages, and potential research insights from various EEG systems

EEG system	Advantages	Disadvantages	Potential research insights
BIS, Sedline, Narcotrend	• Short set-up time • Readily available in most clinical settings • Clinically useful processed EEG metrics • User-friendly	• Limited number and location of electrodes (i.e., only frontal)	• Identifying clinically relevant biomarkers using the raw EEG signal, which can be measured with limited additional time and cost over standard of care procedures
32-Channel Montage	• Ability to collect data from electrode sites across the scalp	• Long set-up time for gel-based systems • Bulky equipment • Cost • Time to train individuals to use the equipment and analyze the output appropriately (including data pre-processing)	• Ability to perform analyses by regions of interest and across the whole head
64 to 256-Channel Montage	• Improved spatial accuracy over ≤ 32 channels • Ability to perform source localization	• Same disadvantages as 32-channel systems • Diminishing returns in terms of spatial accuracy gained vs. cost and computational requirements	• More spatially precise regions of interest and measures of functional interdependence between electrodes • Detecting lateralized brain abnormalities or impairments in postoperative delirium and/or neurocognitive disorder postoperative • Tracking topographical changes in activity or states across time

## Conclusions

In addition to the traditional uses of perioperative EEG monitoring (i.e., to prevent intraoperative awareness, monitor anesthetic “depth” and hasten emergence from anesthesia by decreasing anesthetic agent exposure), an increasing body of work suggests that perioperative EEG data may contain important information on the following: (1) baseline/preoperative brain structure, function, and pathology; (2) postoperative delirium risk; and (3) longer term risk for specific types of ADRD. To harness this information to improve perioperative outcomes, and to identify at-risk patients for delirium prevention interventions and longer term neurologic workup and follow-up, will require close collaboration between neurologists, neuroscientists, neuropsychologists, psychiatrists, geriatricians, surgeons, and anesthesiologists. Research will be necessary to develop new perioperative care models that involve this type of close collaboration, to optimize EEG monitoring devices that can provide necessary data to busy clinicians without slowing down operating room efficiency, and to determine the sensitivity and specificity of intraoperative EEG patterns for various types of ADRD and delirium. Additionally, clinical use of perioperative EEG monitoring to identify patients at risk of delirium and/or ADRD (in the longer term) will necessitate standardization of EEG recording, equipment, and display parameters.

Further, adding EEG monitoring for biomarker detection before, during and after surgery to trials testing interventions to prevent delirium and/or neurocognitive disorder postoperative (whether these interventions are pharmacologic [163, 164] or behavioral [165–167]) provides an opportunity to understand the effects of these interventions on brain network activity and connectivity. Further, EEG monitoring can help clarify whether amelioration of specific delirium-associated EEG patterns is sufficient to prevent delirium itself and associated long-term cognitive impairments or aging-related neurodegenerative disease [168–170]. In this way, perioperative EEG monitoring can help us not only predict or mitigate risk of delirium and neurocognitive disorder postoperative or even overt clinical manifestations of aging-related neurodegenerative diseases, but it can also help us to better understand brain network alterations that may underlie these disorders (and their preclinical manifestations), and to test which of these brain network level processes (i.e., neurophysiologic endophenotypes) may be altered by delirium prevention interventions. Further, EEG could be used to select patients with high delirium risk for inclusion into randomized controlled studies of interventions to prevent delirium.

**Fig. 3:**
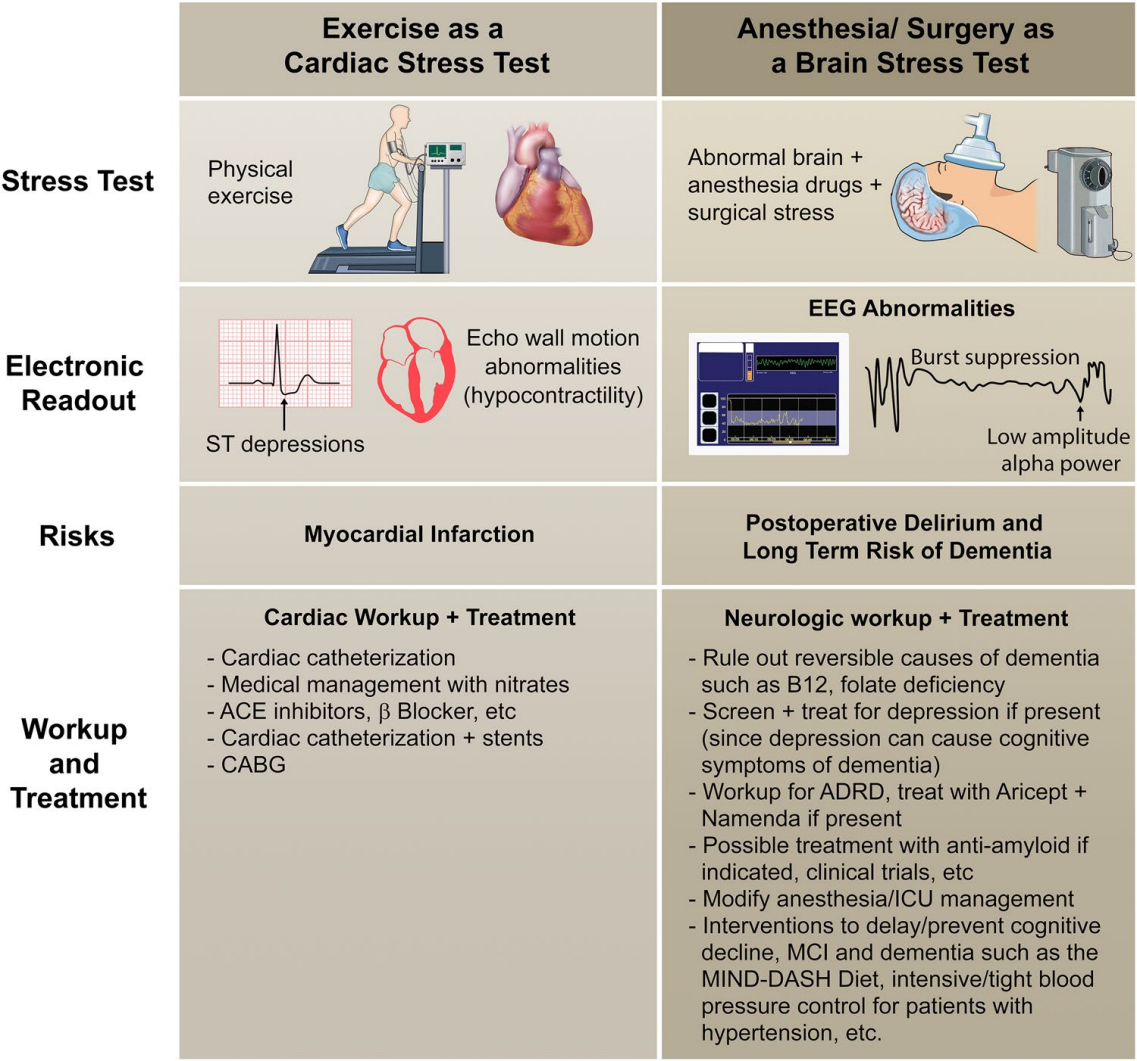
Similarities between the use of ECG in cardiac exercise stress tests, and the potential use of EEG as a real-time readout of patient responses to the stress test of anesthesia/surgery

Much work remains to be done on all of these fronts (see Table 8 for recommended study designs to address these questions). While we do not yet understand how to use EEG monitoring to understand brain structure and function to the same degree as electrocardiograms can be used to provide insight into cardiac structure, function and rhythms, it is important to recognize how much we have learned about the human brain and mechanisms of anesthesia in the last near century since EEG alpha waves were first discovered [171]. This history provides reason to believe that the coming century will witness significant advances in the use of translational perioperative EEG to gather information from the perioperative “stress test” of anesthesia and surgery and to modify care for the benefit of older patients [172], rather than simply waiting for the development of delirium or aging-related neurodegenerative disease in the longer term (Fig. 3).

**Table 8:** Questions for future perioperative EEG research

Question	Potential study designs to address this question
1. What neurologic/neurobiologic mechanisms account for different anesthetic dose adjusted EEG patterns across older adults?	Cohort studies that collect intraoperative EEG, anesthetic dosage data, and measures of brain structure, function, and pathology—i.e., neuroimaging, blood and CSF biomarkers, etc., including multimodal data analysis (e.g., pairing perioperative EEG data with structural or functional brain imaging data) to better localize sources of impairment in postoperative delirium and/or neurocognitive disorder
2. Are there intraoperative anesthetic dose adjusted EEG patterns that indicate specific types of neurodegenerative disease pathology (i.e., amyloid plaques, tau tangles, Lewy bodies, etc.)?	Large cohort studies (potentially multi-center) with intraoperative EEG recording, anesthetic dosage data extraction from electronic medical records, and CSF/blood biomarker and neuroimaging studies for multiple different types of preclinical neurodegenerative disease pathology
3. How can systems of care be designed to refer patients for additional neurologic workup and delirium prevention effort based on perioperative (and anesthetic dose adjusted) EEG patterns?	Feasibility studies of these new care models, followed by implementation science studies to facilitate and operationalize these new care models
4. What preoperative EEG patterns (and anesthetic dose-adjusted EEG patterns) are most predictive of postoperative delirium risk?	Large multi-center cohort studies with intraoperative EEG monitoring, anesthetic dosage data extraction from electronic medical records, and postoperative delirium screening to identify the EEG patterns with the highest sensitivity and specificity for delirium prediction
5. Are there subgroups of patients in whom EEG-guided anesthetic titration will lower postoperative delirium and neurocognitive disorder? If so, what characteristics can be used to identify these subgroups?	Large multi-center randomized controlled trials of EEG-guided anesthetic titration, with deep phenotyping of pre-clinical neurodegenerative disease pathology and neurologic function, and subgroup analyses
6. What intraoperative EEG features (raw or processed measures) are best for using to titrate anesthetic dosage for preventing postoperative delirium and/or neurocognitive disorder?	Large multi-center randomized controlled studies with intraoperative anesthetic titration based on EEG monitoring, in response to EEG patterns identified in #4 and in patients identified in #5 above
7. What should patients (and potentially their family members) be told if they display abnormal intraoperative anesthetic-dose adjusted EEG patterns, or other abnormal perioperative EEG patterns more generally?	Surveys to gauge the feelings and thoughts of patients and family members on this topic, and follow-up studies to examine the effects on families of receiving this information
8. Are there major factors (e.g., anesthetic type and dosage, EEG pre-processing methods, surgery type, hypoactive vs hyperactive delirium type) that influence which preoperative or intraoperative EEG features are best for anesthetic titration or for preventing postoperative delirium and/or neurocognitive disorder?	Large multi-center randomized controlled trials with EEG-guided anesthetic titration, deep phenotyping of pre-clinical and postoperative neurodegenerative disease pathology and neurologic function, and careful analysis of the influence of EEG pre-processing methods (e.g., referencing methods) on conclusions
9. Can we track the fluctuating course of postoperative delirium with EEG to identify brain patterns associated with changes in attentional state among these patients?	Large cohort studies with EEG data collected before, during, and after episodes of postoperative delirium. Studies with 128 + channels could use source localization to identify brain structures that may give rise to these brain activity pattern changes
10. Can we more precisely characterize the types of cognitive impairment (e.g., in different aspects of attention and memory) in postoperative delirium and/or neurocognitive disorder perioperative by utilizing perioperative task-based (event-related) EEG?	Large cohort studies with perioperative EEG collected during neurocognitive attention and/or memory tasks and during stimulus-based EEG event-related potential measurements

## Abbreviations and Definitions

aaMAC: Age-adjusted end-tidal minimum alveolar concentration
AD: Alzheimer’s disease
ADRD: Alzheimer’s disease and/or related dementias
Anteriorization: A spatial shift in EEG power from posterior to frontal
ASA: American Society for Anesthesiology
BIS: Bispectral Index
Coherence: A correlation coefficient for the temporal synchronicity of two brain regions
CNS: Central nervous system
CSF: Cerebrospinal fluid
DARS: Duke Anesthesiology brain Resistance Scale
EEG: Electroencephalography/electroencephalogram
EFNS: European Federation of the Neurological Societies
ETAC: End-tidal anesthetic concentration
ICU: Intensive care unit
Interrelatedness: A general term used to describe the various methods of defining functional coupling or connectivity between two electrodes (or electrode regions)
ISTAART, EPIA: Alzheimer’s Association International Society to Advance Alzheimer’s Research and Treatment, Electrophysiology Professional Interest Area
Lagged linear connectivity: A computed estimate of functional cortical connectivity
LBD: Lewy body dementia
MAC: Minimum alveolar concentration
MCI: Mild cognitive impairment
MRI: Magnetic resonance imaging
NMDA: N-methyl-D-aspartate
PACU: Post-anesthesia care unit
PD: Parkinson’s disease
Phase lag index: A measure of functional connectivity strength that accounts for and removes the effect of volume conduction (distant measurement of an electrical signal from its source, as in EEG scalp electrodes)
PSI: Patient State Index
ROI: Region of interest, as in an area of the scalp measured for its brain region (e.g., frontal, parietal, etc.)
SMC: Subjective memory/cognitive complaints
SR: Suppression ratio
Synchronization likelihood: A method of measuring both linear and non-linear coupling of cortical regions
TIVA: Total intravenous anesthesia
